# Flow regime transitions in flow blurring injection through a CFD parametric study

**DOI:** 10.1038/s41598-025-13047-7

**Published:** 2025-08-01

**Authors:** S. Amirreza S. Madani, Erfan Vaezi, Mohammad Reza Morad, Amir Keshmiri

**Affiliations:** 1 Faculty of Aerospace Engineering, Delft University of Technology, Delft, The Netherlands; 2 Department of Aerospace Engineering, Sharif University of Technology, Tehran, Iran; 3 School of Engineering, The University of Manchester, Manchester, UK

**Keywords:** Flow-blurring, Multiphase flows, Screening analysis, Full factorial design, Computational fluid dynamics, Mechanical engineering, Aerospace engineering, Fluid dynamics

## Abstract

Flow-blurring (FB) is a twin-fluid atomization technique that generates fine sprays through internal turbulent mixing. This study presents a parametric computational investigation of an FB injector operating with air and various liquids at ambient pressure. A validated unsteady two-phase solver based on the Volume of Fluid (VOF) method is used to model the injector at different air-to-liquid mass flow rate ratios (ALRs). Parameters such as penetration length, volume fraction, static pressure, vorticity magnitude, and turbulent kinetic energy are analyzed to understand flow dynamics. The results identify three distinct flow regimes: air-dominant, liquid-dominant, and bubbly flow. Screening analysis of a full factorial design of 32 cases shows that liquid mass flow rate and dynamic viscosity are the most influential factors in penetration length. The resulting penetration length varies between 2 [mm] and 8.5 [mm] across the design space. A correlation analysis confirms these findings and reveals important two-way interactions between parameters, such as the positive effect of combined liquid and air mass flow rates. This insight offers a promising pathway for optimizing flow-blurring injectors in various applications.

## Introduction

**Figure 1:**
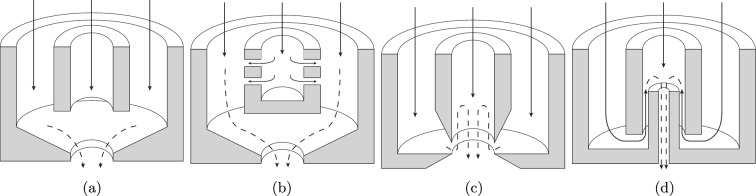
Four standard designs of twin-fluid injectors.

Twin-fluid injection systems use simple atomizer geometries to produce droplets of a relatively uniform size^1^. Some of the applications of these injection systems include gas turbines^2^, combustion^3,4^, spray drying^5^, process industries^6^, and fire suppression^7^. This atomization concept applies secondary flows using the additional momentum of the atomizer flow, i.e. gas phase, to break the liquid jet into ligaments, which further disintegrate into droplets^8^. Figure 1 depicts four standard designs of twin-fluid injection systems. According to the figure, in all designs, fluids are mixed inside the injection system; however, the mixing mechanism differs. In the first design, pressure is used to atomize the liquid into droplets. In the effervescent design, fluids are mixed before being expelled through the nozzle. The flow-blurring design utilizes a high-speed stream of an atomizing gas to generate uniform, small droplets, which is the key point in the performance assessment of atomizers^9^. Lastly, in the counter-flow design, liquid and gas flows are introduced in opposite directions, creating a shear flow that is responsible for breaking the liquid into droplets.

In the case of the flow-blurring design introduced by Gañán-Calvo^10^ in 2005, its advantages resided in its geometrical simplicity, low-cost manufacturing, and uniform spray. Namely, several industries, such as agricultural spraying^11^, cooling purposes^12^, spray combustion^13–15^, and filament production^16–18^, adopted this novel design due to the resulting droplets’ uniform size and velocity distribution. Table 1 shows an overview of applications and the benefits of implementing flow-blurring injection compared with common injections in the mentioned fields.

**Table 1:** Benefits of the flow-blurring spray compared with conventional injectors.

Fields of application	Benefits
Spray cooling	High heat flux removal
	Uniform temperature distribution
	High cooling per unit area
Agricultural spraying	Increase in cooling efficiency
	Uniform distribution pesticide
	Lower chemical pollution
Spray combustion	Increase in droplet size uniformity
	Emission reduction
	Increase in combustion efficiency
Surface coating	Uniform distribution
	Less material consumption
	Low-cost coating process

The twin-fluid atomization process depends on the backflow phenomena inside the liquid tube, resulting the small-scale, two-phase turbulent flows^44^. These flows further generate sprays in specific operating ranges, considering the underlying design provides two injection regimes, including (i) Flow-blurring and (ii) Flow-focusing^10^. This work investigates the atomization physics in the flow-blurring regime underlying the development of design tools for predicting drop size. In this regard, Gañán-Calvo^10^ introduced an experimental relation to calculate a dimensionless drop mass median diameter ($\delta =\text {MMD}/D$; MMD as the mass median diameter and *D* as drop size) from the Weber ($\text {We}=\rho U^2 D/\sigma$, with $\rho$ and $\sigma$ as the fluid density and surface tension, and *U* as the mean droplets velocity), Ohnesorge ($\text {Oh}=\mu /\sqrt{\rho \sigma D}$, with $\mu$ as the liquid dynamic viscosity) and air-to-liquid mass flow rate ratio (ALR) dimensionless numbers as

1 $$\begin{aligned} \delta =0.42\text {We}^{-0.6}(1+18\text {Oh})(1+\text {ALR}^{-1})^{1.2}. \end{aligned}$$

The main focus of this paper is to investigate the two-phase mixing flow physics inside the injection system, which is mainly related to the area near the exit orifice. As far as we know, only three articles have addressed this topic. Agrawal et al.^19^ investigated the effects of ALR, where the atomizer was air, on the spray penetration length of a transparent flow-blurring injector using a high-speed camera. The shadowgraph images allowed observing different mixing flow physics, particularly how an increment of ALR directly affects the penetration length and decreases droplet size.

In another paper, Vardaman et al.^20^ numerically studied the physics of mixing flow near the exit orifice by implementing a RANS-based solver using ANSYS Fluent. Their solver consisted of a two-equation turbulence model coupled with a mixture model. Although they reported that the simulations’ error is not within the acceptable range compared with the experimental data, they reviewed two-phase flow properties, particularly streamlines, pressure distribution, and volume fractions.

Lastly, using numerical and experimental methods, Murugan et al.^21^ characterized the internal flow recirculation along the liquid tube in a scaled-up transparent flow-blurring injector. The experiments provided further insight into the internal mixing and external spray structure for various ALRs ranging from 0.1 to 2 using a high-speed imaging system. The numerical part was devoted to simulating the internal two-phase flow characteristics via a VoF-based LES solver. Then, they compared the penetration length obtained from the numerical simulations with experimental data for different values of ALR to assess the accuracy of the proposed solver. Table 2 summarizes the details of the reviewed papers in the field of the physics of the mixing flow inside the flow-blurring injection system.

**Table 2:** Summary of the reviewed papers in the field of flow-blurring mixing flow.

Paper	Method	Results
Agrawal et al.^19^	High-speed imaging	ALR affects both penetration length and size distribution
Vardaman et al.^20^	RANS simulation	Studying mixing flow properties in terms of ALR
Murugan et al.^21^	High-speed imaging	Proposing a novel solver validated against experiments
	LES simulation	

The present study focuses on the internal flow behavior of flow-blurring (FB) atomizers, with potential applications in spray cooling, combustion, and biomedical aerosol delivery. Unlike previous studies centered on spray breakup and external plumes or often limiting their scope to the impact of the ALR on penetration length, this work emphasizes internal mixing behavior under ambient-pressure conditions. A parametric study is carried out by varying five input factors: liquid density, dynamic viscosity, surface tension, liquid mass flow rate, and air mass flow rate. Moreover, the development of regression models based on corresponding thermofluidic and kinematic parameters remains unexplored. Addressing this gap is necessary as a thorough understanding of the aforementioned factors and their interplay is pivotal for optimizing flow-blurring injection systems, given the significant role of mixing flow plays in determining the flow-blurring spray characteristics. Additionally, the chosen geometric configuration enables the investigation of a wide range of ALR. This framework captures a wide spectrum of operational regimes relevant to practical FB injector design.

**Figure 2:**
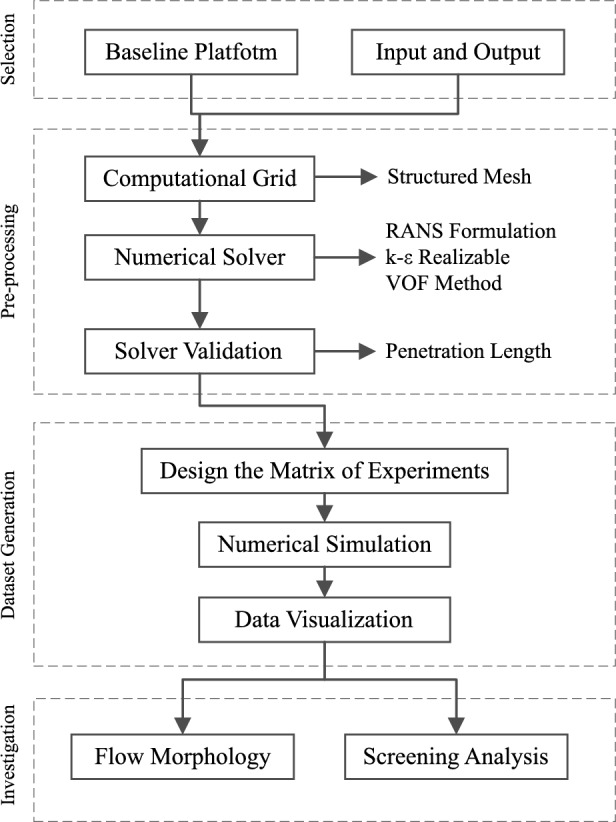
Roadmap of the current research work.

The research roadmap illustrated in Fig. 2 outlines a systematic approach divided into selection, pre-processing, dataset preparation, and investigation. Initially, the inputs and outputs of the flow-blurring spray are delineated in alignment with the study’s objectives. Subsequent steps involve pre-processing, including grid generation, numerical scheme selection, and validation and verification procedures to ensure simulation accuracy. The Full Factorial Design (FFD) method is then employed to construct a standard matrix of experiments. This matrix serves as a foundation for simulations, facilitating data visualization and preprocessing for validation purposes. The final stage involves analyzing the simulation results, focusing on flow morphology and screening analysis to elucidate the underlying dynamics.

This research aims to provide a comprehensive investigation that facilitates a new perspective on flow-blurring atomization processes, improving the understanding of the physics of mixing flow inside the injection system. The main contributions of this work are:

- Conducting an extensive numerical investigation of mixing flows by exploring a wide array of thermofluidic parameters. This detailed examination extends our comprehension of flow dynamics in diverse operational settings, with particular focus on the role of liquid and air mass flow rates, liquid density, dynamic viscosity, surface tension, and their interactions.
- Refining the classification of mixing and two-phase flows by introducing new criteria based on flow characteristics. Our approach enhances the established categories, leading to more precise behavior predictions and design improvements in flow-blurring atomization processes.
- Performing a detailed analysis of primary factors and their two-way interactions using a full factorial design and correlation analysis. This allows for an in-depth understanding of how both main effects and interaction terms influence penetration length. The insights streamline the way for optimization in the design of flow-blurring injectors.
- Validating the findings through a correlation analysis, which quantitatively confirms the intensity of the relationships between the input parameters and their interactions, thus providing a more granular understanding of the mechanisms that dictate the physics of flow-blurring atomization.

The paper is organized as follows: Sections “Baseline platform” and “Methodology” detail the baseline platform and the methodology, including the Computational Fluid Dynamics (CFD) solver and the screening model, while Section “Validation and verification” validates these results against experimental data. Section “Results and discussion” provides an analysis of the flow morphology based on the numerical simulations and illustrates the screening and correlation analysis results, offering new insights into the interaction of key factors. The conclusion is given in Section “Conclusion”, summarizing the study’s contributions, limitations, and proposing future research directions.

## Baseline platform

**Figure 3:**
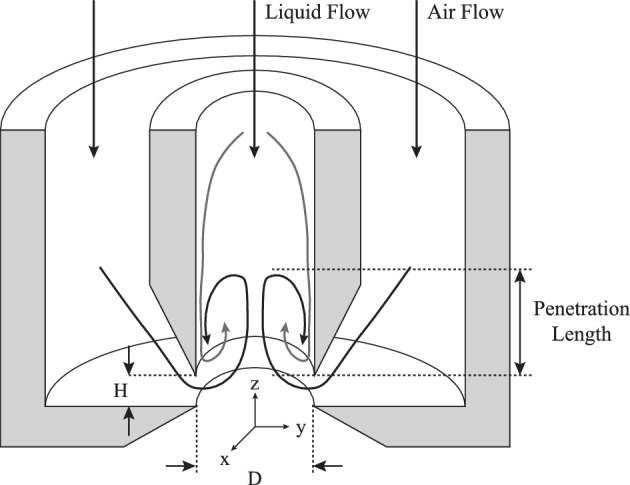
Cross-section of the flow-blurring injection.

The reported efficiency of flow-blurring is higher than that of more complex air-blast atomizers^22^, proposing flow-blurring as an appropriate candidate compared with the other twin-fluid designs^10^. Figure 3 illustrates a cross-section of flow-blurring system, in which *H* and *D* represent the gap between the liquid tube and exit orifice and the diameter of the exit orifice, respectively. In this study $H = 1\,[mm]$ and $D=4\,[mm]$, resulting in $H/D=0.25$.

The internal region where the air and liquid streams first interact and begin to mix is hereafter referred to as the *mixing zone*. As illustrated schematically in Fig. 3, this zone is located just downstream of the inlet junction and encompasses the area where the gas momentum induces backflow, shear-driven recirculation, and bubble formation inside the liquid tube. It extends axially from the base of the orifice to the region where a coherent jet or bubble structure emerges, marking the onset of penetration. Penetration length, as depicted in Fig. 3, is defined along the *z*-axis from the nozzle exit to the axial location where the liquid volume fraction falls below a threshold value of 0.01, corresponding to the end of the continuous liquid phase.

**Figure 4:**
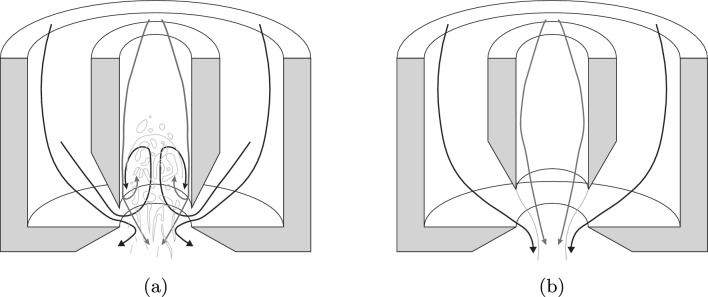
Two regimes in the injection system.

As illustrated in Fig. 4, one can observe the spray if *H*/*D* is less than 0.25 (the design standard value), making it an effective parameter to distinguish between flow-blurring and flow-focusing^10^. In the latter regime, air momentum cannot break the liquid jet, resulting in a thin jet of liquid downstream of the orifice interacting with an airflow bounding it.

The flow-focusing regime can be classified into three classes in terms of instability, including i) the steady jetting regime, ii) the local instability regime, and iii) the global instability regime^23^. On the other hand, in the flow-blurring, the airflow enters the liquid tube to produce backflow and generate bubbles. This leads to thin ligament structures at the nozzle exit, facilitating the atomization process. In this case, after the primary explosive liquid breakup at the nozzle exit, a secondary breakup occurs inside or outside the injector to finalize the process, producing uniform sprays with the required size distributions^24^.

## Methodology

This section discusses the methodology in this work, including the numerical solver and full factorial design.

### Numerical solver

In this study, the flow field inside the injection system is simulated to investigate the characteristics of the penetrating flows using Ansys Fluent^25^. CATIA was used to design the 3D geometry of the injection system and ICEM CFD was subsequently used to generate the computational grid.

The numerical solver is based on the following assumptions: the two-phase flow is unsteady, wall roughness effects are neglected, and gravitational forces are considered. The flow near solid boundaries is modeled using standard wall functions associated with the Realizable *k*–$\varepsilon$ turbulence model. To ensure the validity of this approach, the computational grid was refined such that the first cell adjacent to the wall falls within the logarithmic layer ($30< y^+ < 300$), where the wall function formulation holds. This treatment is widely adopted in two-phase RANS simulations, as direct resolution of the viscous sublayer is not feasible due to the inherent limitations of the turbulence model.

#### Computational grid

**Figure 5:**
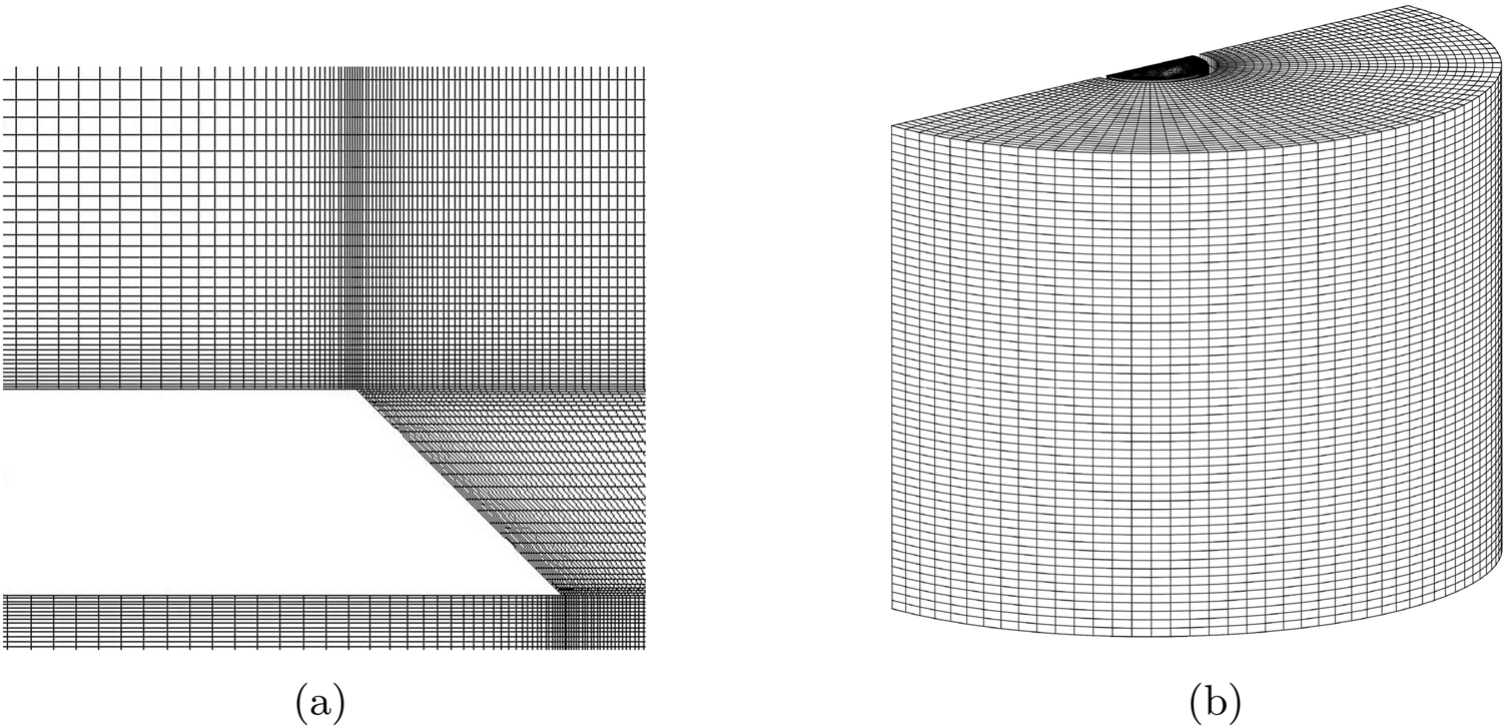
The optimum mesh near the mixing zone and on the control volume.

The computational grid was generated using a structured approach. Figure 5 depicts a cross-section of the applied mesh near the mixing zone and out of the control volume. As shown in the figure, the mesh size is gradually decreased near the mixing zone in order to accurately predict the boundaries of each phase.

The size of the elements is reduced gradually by approaching from the tubes’ inlets to the outlet of the injection system. Details of the optimum numerical grid, including the number of elements and their size, are mentioned in “Validation and verification”. It is noticeable that the final mesh gives $y^+ \le 1$ along all solid boundaries.

#### Governing equations

The three-dimensional formulation of the Reynolds-Averaged Navier-Stokes (RANS) and the Realizable *k*–$\varepsilon$ turbulence model are used to simulate the flow field and turbulence effects on the mean flow, respectively. This is a common approach for simulating a variety of complex flows, particularly two-phase flows with appropriate computational cost^26,27,28,29,30,46,45^.

The Volume of Fluid (VoF) method considers primary and secondary-phase interactions^31^, in which the phases are separated via an indicator function^32^. The model calculates the volume fraction inside control volumes using Eq. 2.

2 $$\begin{aligned} \frac{\partial C}{\partial t} + {\textbf {u}} \nabla C = 0 \end{aligned}$$

where *C*, ${\textbf {u}}$, and *t* are volume fraction, velocity vector, and time, respectively. The model calculates density and dynamic viscosity through Eqs. 3 and 4 as a function of the volume fraction of the primary phase.

3 $$\begin{aligned} \rho =&C\rho _1 + \left(1 -C\right) \rho _2 \end{aligned}$$

4 $$\begin{aligned} \mu =&C\mu _1 + \left( 1-C\right) \mu _2 \end{aligned}$$

Surface tension is modeled using the Continuum Surface Force (CSF) approach proposed by Brackbill et al.^33^, which introduces a volumetric force term in the momentum equation acting along the interface. The surface tension force is computed based on the local interface curvature, which is derived from the gradient of the volume fraction field. As the liquid properties are varied parametrically in this study, the surface tension coefficient $\sigma$ is also updated accordingly for each case to reflect the fluid’s specific interfacial behavior. The momentum equation solved for the multiphase flow is:

5 $$\begin{aligned}\frac{\partial (\rho \textbf{u})}{\partial t} + \nabla \cdot (\rho \textbf{u} \textbf{u}) = -\nabla p + \nabla \cdot \left[ \mu (\nabla \textbf{u} + \nabla \textbf{u}^T) \right] - \nabla \cdot (\rho \textbf{u}' \textbf{u}') + \vec{f}_\sigma + \rho \vec{g}\end{aligned}$$

where $\vec {f}_\sigma$ represents the surface tension force added via the CSF model, and $\vec {g}$ is the gravitational acceleration vector.

#### Boundary conditions

**Figure 6:**
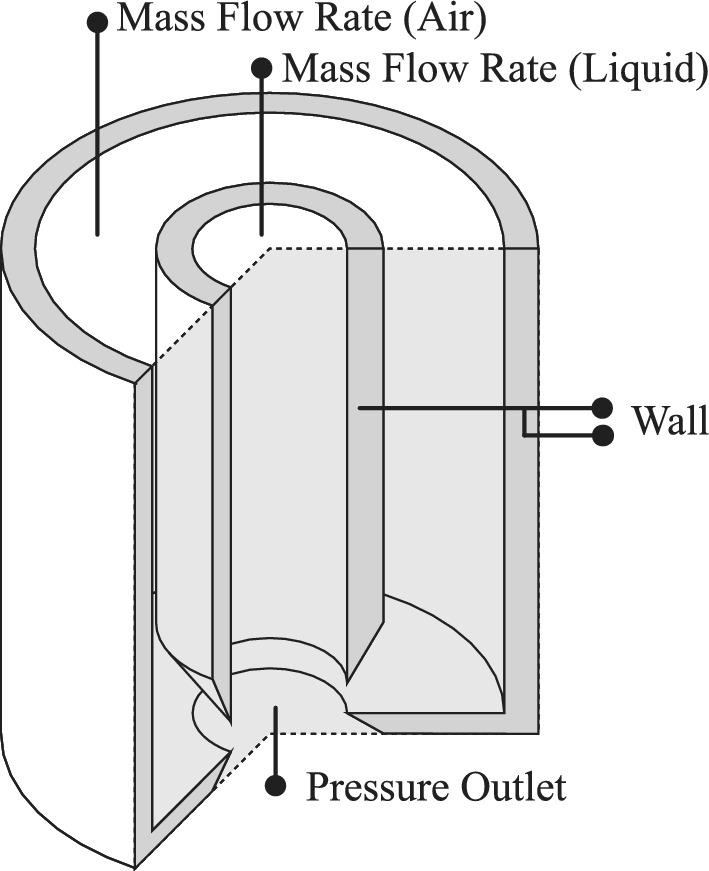
The applied boundary conditions.

Figure 6 illustrates the control volume defined to simulate the two-phase flow. The figure shows liquid and air fluids flowing to the injection system through separate inlets. The total height of the computational domain is selected to ensure that the mixing zone remains well-separated from both the inlets and the pressure outlet boundary. To ensure that the outlet location does not affect the solution, a domain independence test was performed. Results showed that extending the outlet region further changed the penetration length by less than 1.5%, confirming that the domain is sufficiently long.

Furthermore, three different boundary conditions are implemented, including mass flow rate, wall, and pressure outlet. The figure shows that fluids enter the control volume using a mass flow rate boundary condition, separated by adiabatic, no-slip walls. Moreover, the mixed flow exits the control volume through pressure outlet boundary conditions.

#### Numerical scheme

This section briefly discusses the structure of the numerical solver, including time marching criteria, coupling schemes, and discretization methods. Since the physics of penetrating flow is time-dependent, the unsteady solver is required. In this regard, the Courant-Fredrick-Levy (CFL) number is selected to calculate time-step values according to Eq. 6, where *u*, $\Delta t$, and $\Delta x$ are velocity, time-step, and element size, respectively. During solving the flow field, time-step values are selected to satisfy $CFL \le 1/4$.

6 $$\begin{aligned} \text {CFL} = \frac{u\Delta t}{\Delta x} \end{aligned}$$

Regarding the details of the discretization of the governing equations, a SIMPLEC scheme couples pressure and velocity; PRESTO discretizes the pressure, and the least squares cell-based schemes deal with the gradient. The QUICK scheme discretizes the other flow-field variables, which is typical for RANS-based simulations.

### Full factorial design

Statistical methods are reliable tools for investigating and optimizing effective factors in different processes, particularly for defining the matrix of experiments^34^. In this regard, factorial design is one of the most common methods to consider the effects of main factors and multi-way interactions on output parameters^34,35^. In this case, the output is penetration length, representing the physics of mixing flow. Moreover, liquid properties, including density, dynamic viscosity, surface tension, and mass flow rates of liquid and gas, are selected as input factors.

**Figure 7:**
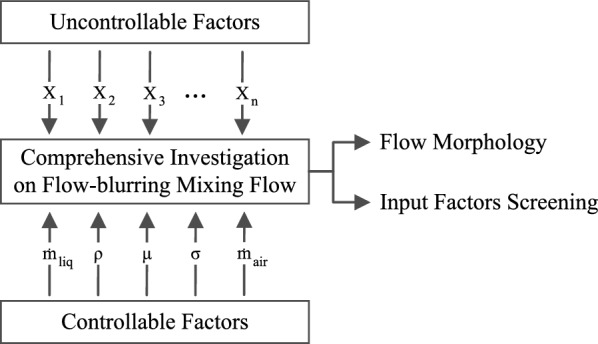
Involved factors in the full factorial design.

Figure 7 illustrates the factorial design used in this study. Factorial designs invariably contend with factors that elude precise control, often due to limitations in measurement tools or inherent inaccuracies. In the context of this research, the primary uncontrollable factors stem from turbulence characteristics, the precision of the solver, and errors associated with spatial discretization.

The purpose of the factorial design is to generate standard datasets for screening the factors following the outputs. Therefore, among the three methods available for the screening process, including the Full Factorial, Fractional Factorial, and Plackett-Burman designs^36^, the full factorial design provides the highest resolution and can study the main effects and their two-way interactions. In contrast, the resolution of other methods is not full by ignoring the effects of factors’ interactions^37^. Hence, the full factorial method is chosen to comprehensively assess effective factors despite a more significant number of runs^38^. The main benefits of the implementation of factorial designs are generating standard matrices of experiments, saving time and expense by minimizing the runs, and detecting uncontrolled factors.

The number of experiments in this method could be calculated as $N_E=L^f$, where $N_E$, *L*, and *f* are the number of experiments, levels, and factors in the matrix of experiments, respectively^39^. Firstly, to build the matrix of experiments, one needs to define the range of input factors. Table 3 details the inputs in this research, where thermofluidic properties belong to water and ethanol.

**Table 3:** Range of thermofluidic and kinematic properties of the liquids and air.

Fluid	Factor	Symbol	Unit	Minimum	Maximum
Liquid	Density	$\rho _{liq}$	[$kg/m^3$]	789	1260
	Dynamic viscosity	$\mu _{liq}$	[*Pa*.*s*]	$1.14\times 10^{-3}$	$7.99\times 10^{-1}$
	Surface tension	$\sigma _{liq}$	[*mN*/*m*]	22.32	63.40
	Mass flow rate	$\dot{m}_{liq}$	[*kg*/*s*]	$2.45\times 10^{-3}$	$1.23\times 10^{-2}$
Air	Mass flow rate	$\dot{m}_{air}$	[*kg*/*s*]	$2.45\times 10^{-3}$	$2.45\times 10^{-2}$

To reduce the impact of uncontrolled disturbance factors (e.g., transient solver fluctuations, memory bottlenecks, and system-level computational noise), the 32 numerical simulations were organized into eight blocks. Each block represents a batch of four simulations executed during a single solver session under consistent computational conditions. This practice, known as *blocking*, is a standard technique in the design of experiments to control for external variability. Blocking ensures that systematic variations in the runtime environment do not bias the results. Furthermore, to randomize potential error propagation, the order of the runs within each block was randomized. This helps distribute the influence of uncontrollable factors evenly across the full factorial matrix, improving statistical robustness of the analysis^40^.

Table 4 illustrates the designed matrix of experiments using the full factorial design method. In the tables, the signs (-) and (+) indicate lower and upper levels of the input factors, respectively.

**Table 4:** Details of defined numerical simulations in the matrix of experiments.

Blocks	#R	Inputs					Treatment Combination
		$\rho _{liq}$	$\mu _{liq}$	$\sigma _{liq}$	$\dot{m}_{liq}$	$\dot{m}_{air}$	
1	1	–	–	–	–	–	1
	2	+	–	–	–	–	$\rho _{liq}$
	3	–	+	–	–	–	$\mu _{liq}$
	4	+	+	–	–	–	$\rho _{liq} \times \mu _{liq}$
2	5	–	–	+	–	–	$\sigma _{liq}$
	6	+	–	+	–	–	$\rho _{liq} \times \sigma _{liq}$
	7	–	+	+	–	–	$\mu _{liq} \times \sigma _{liq}$
	8	+	+	+	–	–	$\rho _{liq} \times \mu _{liq} \times \sigma _{liq}$
3	9	–	–	–	+	–	$\dot{m}_{liq}$
	10	+	–	–	+	–	$\rho _{liq} \times \dot{m}_{liq}$
	11	–	+	–	+	–	$\mu _{liq} \times \dot{m}_{liq}$
	12	+	+	–	+	–	$\rho _{liq} \times \mu _{liq} \times \dot{m}_{liq}$
4	13	–	–	+	+	–	$\sigma _{liq} \times \dot{m}_{liq}$
	14	+	–	+	+	–	$\rho _{liq} \times \sigma _{liq} \times \dot{m}_{liq}$
	15	–	+	+	+	–	$\mu _{liq} \times \sigma _{liq} \times \dot{m}_{liq}$
	16	+	+	+	+	–	$\rho _{liq} \times \mu _{liq} \times \sigma _{liq} \times \dot{m}_{liq}$
5	17	–	–	–	–	+	$\dot{m}_{air}$
	18	+	–	–	–	+	$\rho _{liq} \times \dot{m}_{air}$
	19	–	+	–	–	+	$\mu _{liq} \times \dot{m}_{air}$
	20	+	+	–	–	+	$\rho _{liq} \times \mu _{liq} \times \dot{m}_{air}$
6	21	–	–	+	–	+	$\sigma _{liq} \times \dot{m}_{air}$
	22	+	–	+	–	+	$\rho _{liq} \times \sigma _{liq} \times \dot{m}_{air}$
	23	–	+	+	–	+	$\mu _{liq} \times \sigma _{liq} \times \dot{m}_{air}$
	24	+	+	+	–	+	$\rho _{liq} \times \mu _{liq} \times \sigma _{liq} \times \dot{m}_{air}$
7	25	–	–	–	+	+	$\dot{m}_{liq} \times \dot{m}_{air}$
	26	+	–	–	+	+	$\rho _{liq} \times \dot{m}_{liq} \times \dot{m}_{air}$
	27	–	+	–	+	+	$\mu _{liq} \times \dot{m}_{liq} \times \dot{m}_{air}$
	28	+	+	–	+	+	$\rho _{liq} \times \mu _{liq} \times \dot{m}_{liq} \times \dot{m}_{air}$
8	29	–	–	+	+	+	$\sigma _{liq} \times \dot{m}_{liq} \times \dot{m}_{air}$
	30	+	–	+	+	+	$\rho _{liq} \times \sigma _{liq} \times \dot{m}_{liq} \times \dot{m}_{air}$
	31	–	+	+	+	+	$\mu _{liq} \times \sigma _{liq} \times \dot{m}_{liq} \times \dot{m}_{air}$
	32	+	+	+	+	+	$\rho _{liq} \times \mu _{liq} \times \sigma _{liq} \times \dot{m}_{liq} \times \dot{m}_{air}$

## Validation and verification

Firstly, a mesh study allows for finding the optimum computational grid regarding the number of elements and computational time. Secondly, the penetration length is quantitatively compared with the available experimental data. To do so, we replicated the injection system studied by Vardaman et al.^20^, which exactly parallels the case study of our current work. We specifically focused on the case with an ALR of 2.0, utilizing water and air as the working fluids with mass flow rates of $1.23\times 10^{-3}$ [*kg*/*s*] and $2.45\times 10^{-3}$ [*kg*/*s*], respectively.

The penetration length is defined as the axial distance from the nozzle exit to the point where the liquid volume fraction first drops below 0.01. This quantity is extracted from the time-averaged volume fraction field of the liquid phase. Simulations are advanced with a variable time step governed by a CFL condition, and flow statistics are sampled only after the initial transient has passed. The averaging window spans the final portion of each run (typically the last 20–25% of the physical simulation time), during which the flow exhibits statistically steady behavior. Convergence of the time-averaged penetration length was verified by comparing successive moving averages, which varied by less than 1%, confirming the reliability of the sampling strategy.

### Mesh study

**Figure 8:**
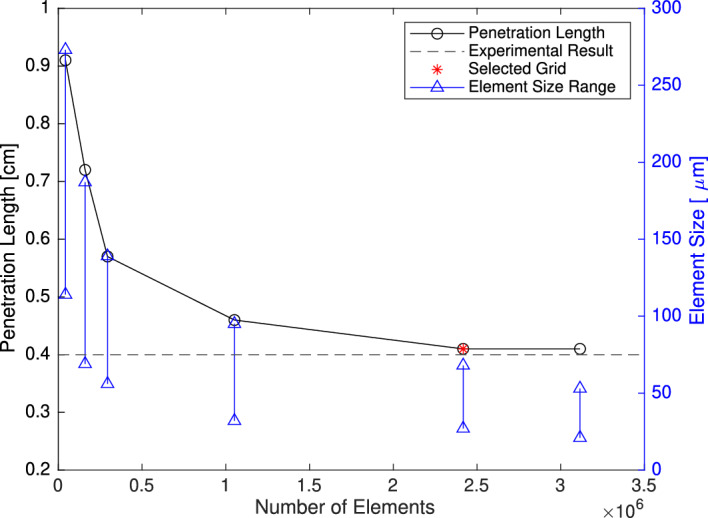
Penetration length vs. the number of elements and grids’ minimum and maximum element size.

Figure 8 illustrates the diagram of penetration length as a function of the number of elements and grid size range. The minimum and maximum size of elements depend on the cubic root of the elements’ volumes. The coarser grids overestimate the experimental data for the penetration length; however, by refining the grid size, particularly in the mixing region, the solution gradually converges to a valid value at the grid with 2.4 million elements, selected as the optimum, considering accuracy and computational cost.

### Parameter comparison

Since the penetration length is a crucial parameter in this work, it is used to validate the numerical solver. Therefore, the proposed results are compared to the experimental data for various ALRs. Table 5 compares the numerical results of the current work and the numerical and experimental results of Vardaman et al.^20^. Also, the corresponding error percentages against the experimental result of Vardaman et al.^20^ are demonstrated. The accuracy of the simulations is higher as a result of optimizing both the numerical scheme and computational grid. The maximum error corresponds to the ALR of 0.8 in both solvers, reduced to less than $20\%$ from almost $233\%$. Moreover, the minimum error decreased to $2.8\%$ compared to $81\%$ in^20^.

**Table 5:** Penetration length values [*mm*] reported in the current work and reference paper^20^.

Parameter	ALR	Vardaman et al.^20^		Current Work
		Experimental	Numerical	Numerical
$P_L$ [*mm*]	0.8	1.5	5	1.8
	1.0	3.2	5.8	3.5
	1.5	3.6	7.2	3.7
	2.0	4.4	8.2	4.0
Error [%]	0.8	N/A	233.3	19.95
	1.0	N/A	81.25	9.37
	1.5	N/A	99.98	2.78
	2.0	N/A	86.36	9.01

## Results and discussion

In the initial segment of this section, the mixing flow within the flow-blurring injector is categorized into three regimes based on the fluid flow characteristics. This is followed by an exploration of screening models through the Full Factorial Design method. The analysis considers the main effects of the inputs, which pertain to the influence of each independent variable on the dependent variable (output), as well as the two-way interactions. These interactions are the combined effects of pairs of independent variables on the dependent variable, where the impact of one independent variable is influenced by the level of another.

The flow field inside the flow-blurring injector is assumed to be homogeneous in the radial direction, meaning that the variation of flow properties along the angular coordinate $\theta$ is negligible. Mathematically, this homogeneity can be expressed as

$$\begin{aligned} \frac{\partial \phi }{\partial \theta } \approx 0, \end{aligned}$$

where $\phi$ represents any flow variable (e.g., velocity, pressure, etc.). Physically, this implies that flow characteristics at any angular position remain consistent, allowing us to average the flow data over the angular direction. As a result, the following results are averaged values in the angular direction.

As depicted in Fig. 3, the data is processed using a cylindrical coordinate system, where the axial direction corresponds to the $z$-axis, and the radial and angular directions lie on the $xy$-plane. The injector’s primary axis is aligned with the $z$-axis, ensuring that the main flow propagates axially. This approach follows previous studies, where high-speed visualizations and numerical simulations confirmed the homogeneity of the flow within the injector (see^22,41,42,43^).

All flow statistics are computed over a time-averaging window that begins after the initial transient phase. The simulation uses a variable time step governed by a CFL condition, and data sampling for volume fraction and velocity fields begins once the flow reaches statistical stationarity. The averaging window spans the last 20–25% of each run’s physical time, during which 200–300 field snapshots are recorded. Convergence was confirmed by comparing moving averages, with variation below 1%.

### Flow morphology

**Figure 9:**
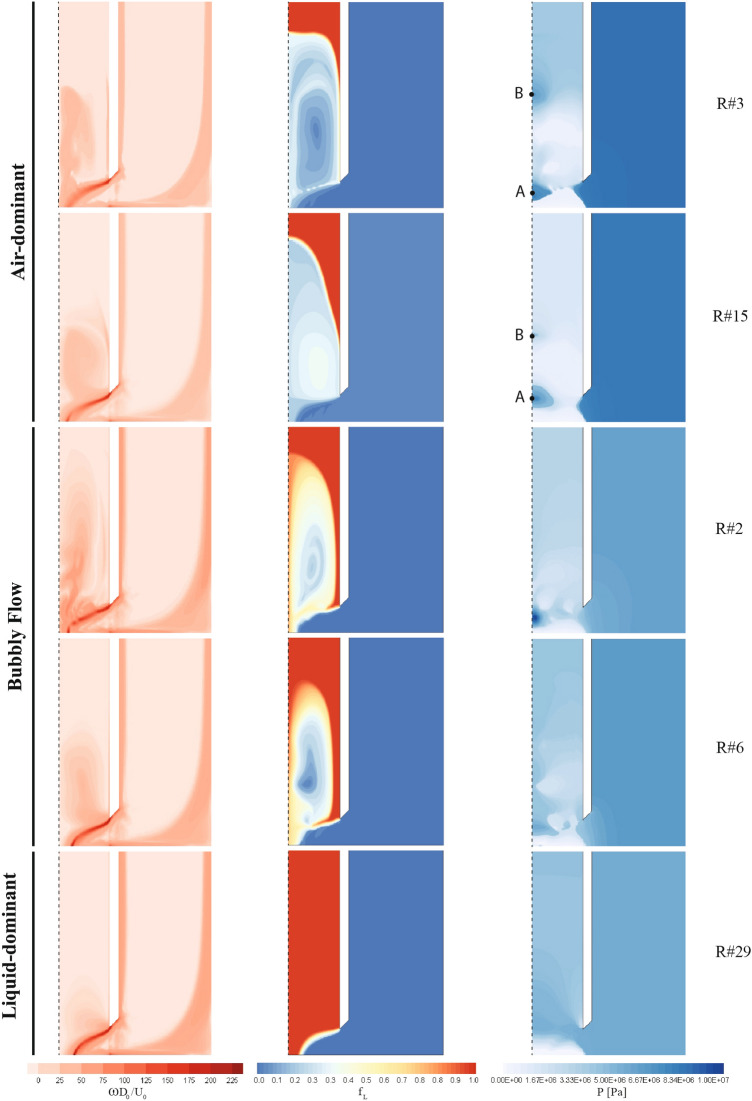
Vorticity magnitude (left), liquid volume fraction (middle), and static pressure distribution (right), R numbers given on the right-hand side of the contours representing different runs in the matrix of experiments, as well as points A and B indicate stagnation points.

Figure 9 presents the vorticity magnitude, volume fraction, and static pressure distribution on the averaged control volume in the homogeneous direction. These contours illustrate the different flow properties and patterns across the observed regimes, particularly within the liquid tube and the mixing zone near the outlet. It is notable that the air tube remains largely unaffected by the two-phase interactions.

The vorticity magnitude contours, as shown in the left column, reveal the rotational intensity of the flow across the regimes. In the air-dominant regime, the vorticity levels are higher due to the strong momentum of the air, particularly near the boundaries of the liquid tube. As the flow transitions to the bubbly flow regime, the vorticity is more dispersed, with distinct vortices generated by bubble dynamics. In contrast, the liquid-dominant regime exhibits lower vorticity, reflecting the weaker penetration of the atomizer’s airflow and the lack of significant mixing at the interface.

Based on the volume fraction contours, the distribution of the liquid phase exhibits distinct and regime-specific characteristics. In air-dominant cases (e.g., R#3 and R#15), the air jet penetrates deep into the liquid tube, producing elongated, dispersed structures and sharp liquid–gas interfaces near the outlet. In bubbly-flow regimes (e.g., R#2, R#6), the interface is highly fragmented, with significant recirculation zones and interfacial instabilities visible within the mixing region. Conversely, in liquid-dominant cases (e.g., R#29), the liquid core remains largely intact, and the gas fails to form significant penetration, resulting in a blunt, compact volume fraction profile. These differences confirm that the volume fraction field provides a clear indicator of regime identity and internal mixing efficiency.

- **Air-dominant:** The strong momentum of the airflow enables it to penetrate the liquid tube with high volume fraction values, generating a nearly uniform spray structure with fine droplets at the outlet. The boundary between the two phases oscillates over time, though the air remains dominant.
- **Bubbly flow:** Air momentum also penetrates the liquid tube, but instead of dominance, a balanced state is reached. The formation of bubbles is a defining feature of this regime. The transient nature of bubble dynamics leads to temporal variations in flow properties.
- **Liquid-dominant:** In this regime, the atomizer’s momentum is too weak to penetrate the liquid tube. The primary breakup of the liquid is minimal, and the air flow mainly serves to reduce the jet radius. Significant gradients in flow properties can be observed along the thin boundary between the liquid and air phases.

The static pressure contours, depicted in the right column of Fig. 9, show uniform pressure distribution within the air tube, unaffected by variations in the flow regime. However, the pressure within the liquid tube is influenced by the mixing dynamics. In the air-dominant regime, two stagnation points are identified: one near the outlet (A) and a second upstream in the liquid tube (B), fluctuating with time. The bubbly flow regime exhibits non-uniform stagnation points due to bubble-induced flow fluctuations, with the most prominent one located within the mixing zone. In the liquid-dominant regime, no stagnation points are present as air momentum is insufficient to significantly alter the liquid’s flow. The liquid-dominant regime thus experiences the most uniform pressure distribution compared to other regimes.

**Figure 10:**
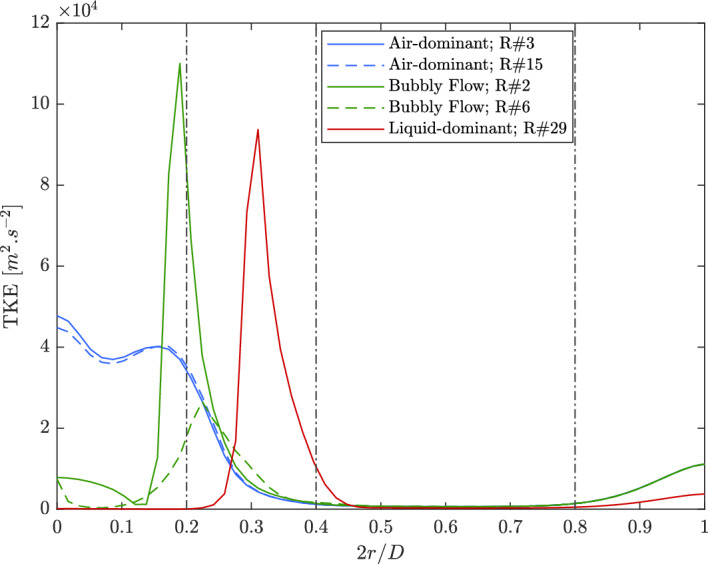
Turbulent kinetic energy profiles along the orifice.

The turbulent kinetic energy (TKE) profiles at the injector outlet are illustrated in Fig. 10. The profiles are segmented into four distinct regions based on the dimensionless radial position $2r/D$, where $r$ represents the radial distance and $D$ is the orifice diameter. The regions considered are: $0.0 \le 2r/D \le 0.2$ (section 1), $0.2 < 2r/D \le 0.4$ (section 2), $0.4 < 2r/D \le 0.8$ (section 3), and $0.8 < 2r/D \le 1.0$ (section 4).

- **Section (1):** This region displays the most significant differences between the air-dominant, bubbly, and liquid-dominant regimes. In the air-dominant regime, TKE is highest near the centerline ($2r/D \approx 0.1$), where the mixing between air and residual liquid generates strong turbulent interactions. Two local maxima are observed in this section due to recirculation and mixing zones. For the bubbly flow regime, a similar peak in TKE occurs near $2r/D = 0.2$, indicating strong interactions between phases at this boundary. However, in the liquid-dominant regime, TKE remains significantly lower near the centerline, reflecting the dominance of the liquid core, where phase interactions are minimal. The increase in TKE near $2r/D \approx 0.2$ suggests that the high-momentum air surrounding the liquid jet begins to interact with the liquid, increasing turbulence at the interface.
- **Section (2):** This section shows a sharp drop in TKE for all regimes as the phase interactions reduce beyond $2r/D = 0.2$. However, the liquid-dominant regime exhibits a pronounced increase in TKE at $2r/D \approx 0.3$, corresponding to the point where the air momentum increases significantly as it moves around the liquid core. This increase highlights the role of the air phase in generating turbulence at the interface between the liquid and air. For the air-dominant and bubbly regimes, TKE decreases steadily.
- **Section (3):** TKE remains relatively constant in this region for all regimes. This suggests that beyond $2r/D \approx 0.4$, the flow becomes more stabilized with minimal phase interactions. The air is primarily responsible for the flow behavior, and the absence of significant circulations keeps TKE at its lowest level compared to the other regions.
- **Section (4):** In this final section, all regimes experience a local increase in TKE near $2r/D = 0.8$. This increase is attributed to the interactions between the flow and the outlet walls, which cause recirculations and increased turbulence. The liquid-dominant regime shows the least slope in this region, indicating that the liquid’s presence continues to suppress turbulence compared to the air-dominant and bubbly flow regimes. The TKE in the air-dominant and bubbly regimes is much higher, as these flows are driven by air momentum, leading to stronger circulations near the walls.

**Figure 11:**
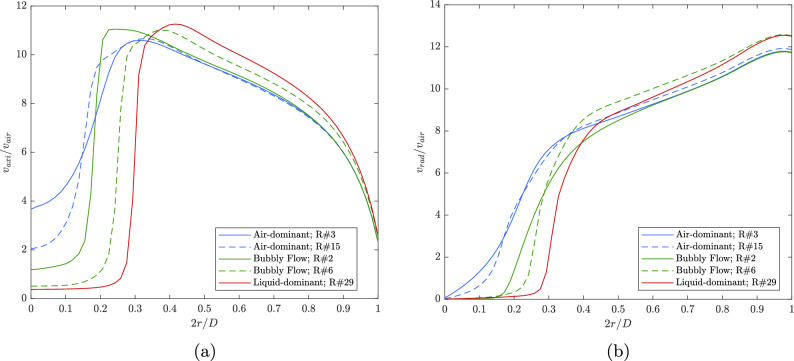
Radial and axial velocity along the orifice.

Figure 11 presents the normalized axial and radial velocity profiles at the injector’s orifice outlet, averaged over the angular direction. The velocities are normalized by the corresponding incoming air velocity, $v_{\text {air}}$, and plotted as a function of the dimensionless radial position $2r/D$, where $D$ is the orifice diameter. The profiles represent the air-dominant, bubbly flow, and liquid-dominant regimes, and each flow regime exhibits distinct characteristics.

In Fig. 11a, the normalized axial velocity $v_{\text {axial}}/v_{\text {air}}$ is shown. For the air-dominant regime, there is a sharp rise in axial velocity near the centerline ($2r/D \approx 0.2$), followed by a peak and then a gradual decline toward the outer radius. This indicates that the air primarily accelerates in the axial direction with a strong central influence. The bubbly flow regime shows a similar trend but with a slightly higher peak, indicating that the presence of liquid reduces the axial momentum of the air, leading to a broader velocity profile near the centerline but reaching higher values for $2r/D \gtrsim 0.2$.

The liquid-dominant regime exhibits a distinctive behavior. The axial velocity remains constant at a low value near the centerline, up to approximately $2r/D \approx 0.2$, after which there is a sharp increase as the axial velocity transitions to the region dominated by the surrounding high-momentum air flow. This behavior reflects the fact that in this regime, the liquid jet is confined near the centerline, and the surrounding high-speed air accelerates as it flows around the liquid. As the radial position increases, the axial velocity gradually decreases again. Notably, while the axial velocity remains lower near the centerline compared to the air-dominant regime, it becomes higher at larger radial positions as the air flows around the liquid core.

Figure 11b shows the normalized radial velocity $v_{\text {radial}}/v_{\text {air}}$. In the air-dominant regime, the radial velocity increases sharply as the flow expands outward, peaking near the edge of the orifice. This increase is damped moving across the orifice. The bubbly flow regime shows a more moderate radial velocity increase due to the interaction between air and liquid, which moderates the radial expansion. The liquid-dominant regime, however, presents a different pattern. While the radial velocity is low near the centerline, it increases significantly at larger radial positions as the air moves around the liquid jet, indicating that the liquid jet’s influence confines the flow near the center, while the surrounding high-momentum air escapes radially.

**Figure 12:**
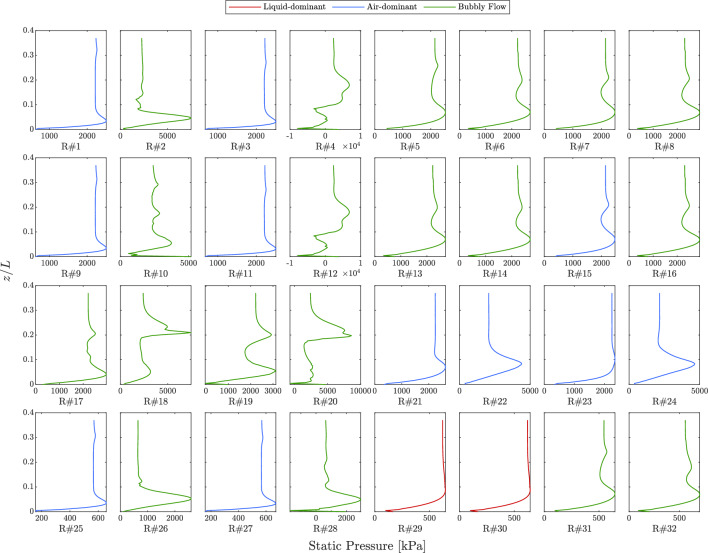
Static pressure distribution along the central axis for all simulations of the matrix of experiments.

Figure 12 illustrates the distribution of static pressure along the central axis of the injector system for all 32 numerical simulations, which were conducted as part of the matrix of experiments. These simulations have been categorized based on their corresponding flow features. The static pressure distribution is shown for the range $0< z/L < 0.4$, where $L$ is the total height of the injector. No significant changes were observed for $0.4< z/L < 1$ across all simulations, so only the lower portion is displayed. This representation emphasizes the distinct behaviors of static pressure along the central axis for different flow regimes, facilitating classification based on schematic patterns and flow characteristics.

- **Air-dominant:** In this regime, the pressure curves have a similar overall shape but vary in terms of values. The static pressure exhibits a smooth and steady decline along the central axis, due to a singular phase transition between the liquid and air inside the injector. This pattern persists until approximately $z/L_0 = 0.1$, after which the pressure curves reach a local extremum within the range $0.05< z/L < 0.1$, marking a stagnation point, labeled as point A in Fig. 9. Another stagnation point (point B) is observed around $z/L \approx 0.3$ in simulations 1, 3, 9, 11, 15, 25, and 27, reflecting a temporary flow recirculation zone. Finally, near the outlet, the static pressure decreases significantly.
- **Bubbly Flow:** The static pressure profiles in this regime show irregularities and fluctuations along the axis, attributed to the transient and stochastic nature of bubble generation. In simulations 5-8, 13, 14, 16, 28, 31, and 32, the static pressure curves resemble those of the air-dominant regime but with additional fluctuations before reaching their extrema. For the other bubbly flow simulations, the curves exhibit a series of relative minima and maxima along the axis, reflecting the turbulent interactions and recirculations caused by the generation of bubbles. Some of these simulations also show a drop in pressure near the outlet.
- **Liquid-dominant:** The pressure profiles for the liquid-dominant regime are the smoothest among the three, as no significant phase gradient exists along the central axis. The diagrams for this regime are nearly identical, both in terms of their schematic shape and values. No significant extrema are observed along the axis, and the pressure curves exhibit a rapid decline beginning around $z/L = 0.1$. This pattern reflects the dominance of liquid in this regime, resulting in a relatively consistent pressure drop along the axis, without the fluctuations seen in the other regimes.

### Screening analysis

The screening analysis aims to find the effectiveness of input factors on the penetration length, being validated in terms of R-squared and error distributions before proposing the model.

**Figure 13:**
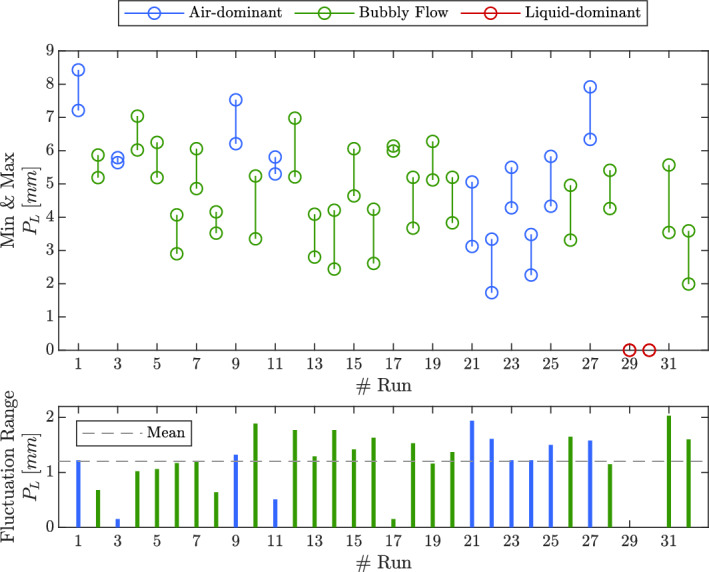
Distribution of instantaneous penetration length values and the fluctuation range in terms of run numbers.

The standard structure of the matrix and wide range of inputs allow for observing dominant physical phenomena and patterns. Figure 13 shows the instantaneous penetration length’s maximum and minimum values and the fluctuation range for each numerical simulation. These values are extracted from 200–300 unsteady snapshots, depending on the simulation time step evolution. Owing to the fact that the simulations were in a transient state, each figure fluctuated between its minimum and maximum value in an average 1.2 [*mm*] range. The different values included in the figure are based on the standard structure of the matrix of experiments. Since most values range between 3 and 6 [*mm*], there are enough samples for screening purposes. It is noticeable that there are two numerical simulations that depict no penetrating flow inside the liquid tube, which, as discussed earlier, are labeled as liquid-dominant regimes.

#### Model performance evaluation

Table 6 shows the R-squared values related to the screening model. The table provides three types of R-squared values: overall, adjusted, and predicted, which are calculated to assess the accuracy of the model. According to the table, the lowest accuracy is associated with the predicted R-squared, while the highest accuracy is indicated by the overall R-squared value. Overall, the R-squared values confirm the reliability of the model based on the screening analysis.

**Table 6:** R-squared values of the screening model.

R-squared Type	Overall	Adjusted	Predicted
Value	93.5%	92.7%	90.8%

**Figure 14:**
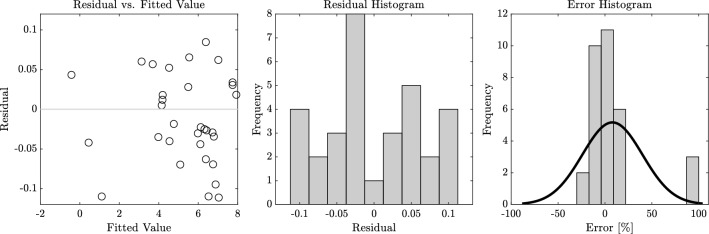
Performance evaluation plots of the screening model.

Figure 14 presents residual plots related to the screening model, including the residual vs fitted value plot, residual histogram, and error percentage histogram. These plots offer insights into the model’s performance by highlighting discrepancies between predicted and actual values.

In the residual vs fitted value plot, the residuals are scattered evenly above and below the zero line, indicating that the model’s errors are randomly distributed and not biased toward over- or under-prediction. The residual histogram further illustrates the spread of the residuals, which are centered around zero, demonstrating that most predictions are accurate with small deviations. Lastly, the error percentage histogram shows that the majority of errors are clustered near 0%, confirming that the model predictions align closely with the actual values. The fitted Gaussian normal distribution in the error histogram supports this, with its peak close to zero, indicating a well-performing model.

#### Main effects

**Figure 15:**
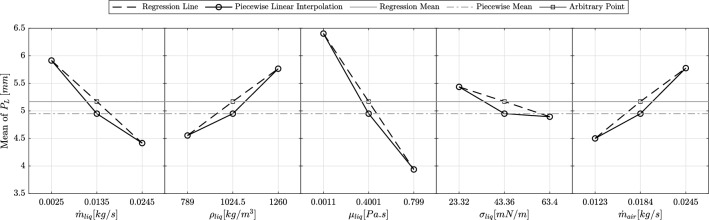
Main effects of the input factors on the penetration length.

As mentioned, five factors are selected as inputs to analyze their effect on the penetration length. This subsection discusses the main effects by evaluating their general trends and magnitudes. Figure 15 shows the main effects of these input factors on penetration length, based on the range of each factor as outlined in Table 3. To ensure the accuracy of the regression analysis, the center point of the experimental matrix is included, showing a 4.44% difference between the regression and piecewise mean values.

According to the figure, both liquid density and air mass flow rate have a direct positive effect on the penetration length, meaning that increasing these factors results in a longer penetration length. In contrast, the other three factors-liquid mass flow rate, dynamic viscosity, and surface tension-exhibit an inverse relationship with penetration length, as higher values of these parameters lead to a shorter penetration length.

The slopes of the lines provide insight into the relative influence of each input factor. As depicted, the liquid’s dynamic viscosity is the most influential factor, showing the steepest slope. This suggests that small changes in viscosity can lead to significant variations in penetration length. On the other hand, surface tension has the least effect, as indicated by its nearly flat slope. Interestingly, liquid density and air mass flow rate exhibit similar effects on both the direction and magnitude of the penetration length variation, as their slopes are comparable.

#### Two-way interactions

**Figure 16:**
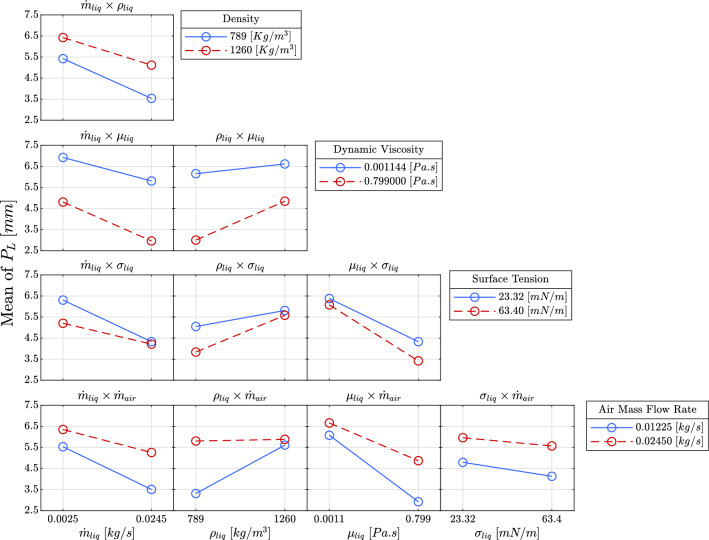
Two-way interactions of the input factors on penetration length.

Figure 16 illustrates the effects of two-way interactions between input factors on penetration length. These interactions, represented as binary products of the input factors, provide a deeper understanding of the underlying flow physics and enable the prediction of mixing behavior when two factors vary simultaneously. The two-way interactions are grouped into four categories based on the input factors.

Firstly, the interaction between the liquid mass flow rate and other factors shows a consistent inverse relationship with penetration length across all cases. Regardless of the combination, increasing the liquid mass flow rate results in a reduction of penetration length. This is because a higher mass flow rate increases the liquid supply, causing the injected liquid to spread out more and reducing its penetration. Additionally, this behavior is more pronounced when the liquid viscosity is low, as seen in the respective diagrams.

For liquid density interactions, there is a direct correlation with penetration length. As liquid density increases, so does the penetration length, owing to the higher momentum imparted to the liquid jet due to its increased mass. Notably, the interaction between liquid density and air mass flow rate shows minimal influence at higher air mass flow rates. The nearly flat slope in this case suggests that the effect of liquid density diminishes as air momentum becomes the dominant factor.

The interactions involving liquid viscosity have a pronounced inverse effect on penetration length. As viscosity increases, the fluid’s resistance to deformation intensifies, leading to slower flow and reduced penetration length. The widening disparity between lines in these plots indicates that as viscosity increases, the influence of other factors becomes less significant. This suggests that viscosity is a controlling factor in penetration dynamics, especially when the liquid’s internal resistance dominates the flow behavior.

Lastly, the interaction between surface tension and air mass flow rate exhibits the least effect on penetration length. Surface tension primarily affects the breakup of the liquid jet rather than its initial penetration. Hence, its influence on the macroscopic penetration length remains limited, especially in comparison to other factors. Furthermore, it is important to note that mass flow rate and liquid density are independent variables. While mass flow rate is dependent on velocity, liquid density is an inherent property of the working fluid, contributing to the overall momentum but not directly influencing the mass flow rate.

**Figure 17:**
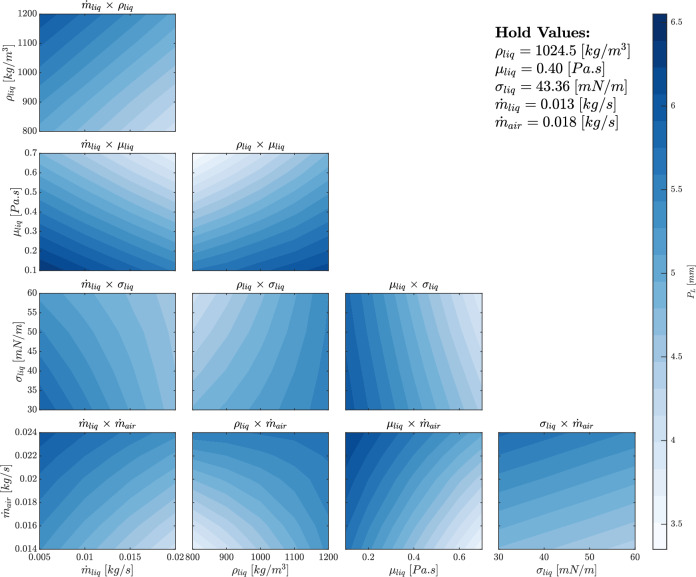
Contour plots of the two-way interaction effects of the inputs on the penetration length.

Figure 17 presents contour plots of the two-way interaction effects between input parameters on penetration length. The interaction plots reveal how pairs of input factors combine to affect the penetration length, with the remaining factors held constant at their average values. The patterns observed in these contour plots offer insight into the relative influence of each parameter and their combined effects on the penetration length.

For the $\dot{m}_{liq} \times \rho _{liq}$, $\dot{m}_{liq} \times \mu _{liq}$, and $\dot{m}_{liq} \times \sigma _{liq}$ plots, the liquid mass flow rate $\dot{m}_{liq}$ consistently shows an inverse relationship with penetration length. This is because a higher liquid flow rate increases the overall liquid volume, leading to broader spreading rather than deeper penetration. Liquid density $\rho _{liq}$ and viscosity $\mu _{liq}$ both exhibit significant impacts when interacting with $\dot{m}_{liq}$, with liquid viscosity having the strongest effect due to its role in resisting flow, as shown by the steeper contour slopes. Surface tension $\sigma _{liq}$, while still inversely related to penetration length, exhibits a weaker influence compared to viscosity.

In the interaction plots involving air mass flow rate $\dot{m}_{air}$, such as $\dot{m}_{liq} \times \dot{m}_{air}$ and $\rho _{liq} \times \dot{m}_{air}$, increasing $\dot{m}_{air}$ leads to a direct increase in penetration length. This is due to the air’s contribution to the overall momentum of the flow, which dominates when combined with the liquid mass flow rate or density. Interestingly, both $\dot{m}_{air}$ and $\rho _{liq}$ have similar influences, as indicated by the comparable spacing of contour lines, though $\dot{m}_{air}$ tends to have a slightly greater effect when compared directly with other parameters.

For interactions involving viscosity, such as $\rho _{liq} \times \mu _{liq}$ and $\mu _{liq} \times \dot{m}_{air}$, higher viscosity reduces penetration length significantly, with the contour lines showing steep gradients. This indicates that viscosity’s influence becomes more dominant when combined with other parameters like air mass flow rate, which further affects the fluid’s ability to penetrate deeper into the medium. Conversely, interactions involving surface tension, such as $\sigma _{liq} \times \dot{m}_{air}$, exhibit weaker effects, with surface tension having a relatively minor role in determining penetration length compared to viscosity and mass flow rate.

To wrap up with, while air mass flow rate and liquid viscosity emerge as the most influential parameters affecting penetration length, other parameters like liquid density and surface tension also contribute but to a lesser extent. The contour plots illustrate how combinations of parameters either enhance or diminish penetration, with air momentum and fluid resistance playing key roles.

### Correlation analysis

To complement the screening study, a correlation analysis was performed to evaluate the linear relationship between each input factor and the penetration length. Pearson correlation coefficients $r$ were computed for all parameters across the 32 cases in the design matrix using the standard definition:

7 $$\begin{aligned} r = \frac{\sum _{i=1}^N (x_i - \bar{x})(y_i - \bar{y})}{\sqrt{\sum _{i=1}^N (x_i - \bar{x})^2} \sqrt{\sum _{i=1}^N (y_i - \bar{y})^2}} \end{aligned}$$

where $x_i$ and $y_i$ are the values of an input variable and the corresponding penetration length, respectively, and $N$ is the total number of cases. A positive $r$ indicates a direct correlation, while a negative value implies an inverse trend.

**Figure 18:**
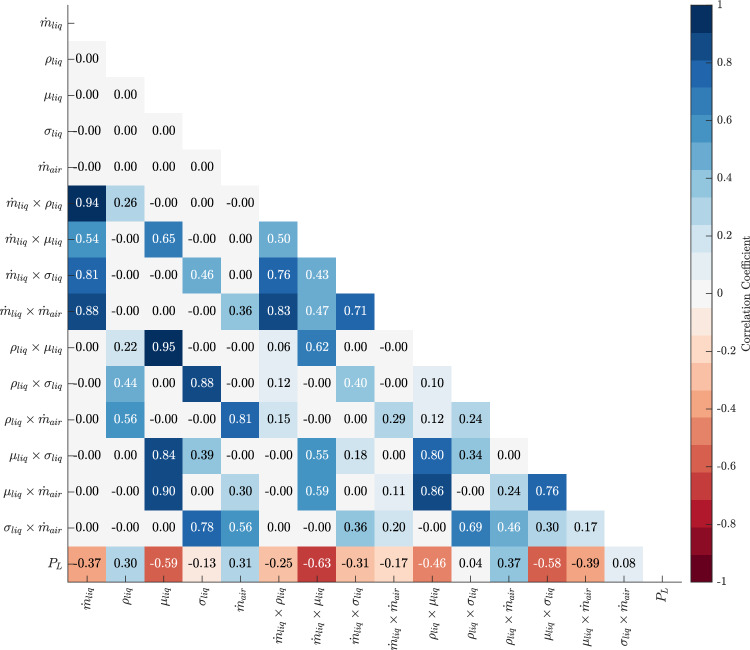
Correlation map of the main effects and two-way interactions.

Figure 18 illustrates the correlation analysis for the main effects and two-way interactions, providing a quantitative understanding of the relationship between input factors and penetration length, $P_L$. This analysis complements the earlier screening analysis, enabling us to pinpoint not only which factors are most influential but also the intensity of their effects on the output variable.

As observed, the liquid mass flow rate $\dot{m}_{liq}$ shows a moderate negative correlation with $P_L$ ($-0.37$), confirming its inverse relationship as identified in the screening analysis. This highlights that an increase in liquid mass flow rate leads to a decrease in penetration length. Similarly, dynamic viscosity $\mu _{liq}$ exhibits a strong negative correlation with $P_L$ ($-0.59$), further emphasizing its role as a dominant factor in reducing penetration length by increasing the resistance to flow, consistent with previous findings.

Other key input parameters, such as surface tension $\sigma _{liq}$, show weaker correlations with $P_L$ ($-0.13$), indicating that its effect is less pronounced compared to other factors. Air mass flow rate $\dot{m}_{air}$, on the other hand, displays a positive correlation ($0.31$), reinforcing its role in increasing penetration length by enhancing atomization and momentum transfer, as previously suggested by the screening analysis.

This analysis also provides insight into the two-way interactions between input variables. The interaction between liquid mass flow rate and air mass flow rate ($\dot{m}_{liq} \times \dot{m}_{air}$) shows a moderate positive correlation with $P_L$ ($0.71$), indicating that increasing both factors leads to enhanced penetration. Similarly, the interaction between dynamic viscosity and air mass flow rate ($\mu _{liq} \times \dot{m}_{air}$) has a significant positive correlation ($0.76$), implying that while viscosity alone tends to reduce penetration, its combination with a high air mass flow rate can counterbalance this effect and increase penetration length.

In contrast, the interaction between liquid mass flow rate and dynamic viscosity ($\dot{m}_{liq} \times \mu _{liq}$) shows a strong negative correlation with $P_L$ ($-0.63$), suggesting that higher liquid flow rates coupled with increased viscosity significantly reduce penetration length due to the compounded resistance to flow.

Overall, this correlation analysis validates the results of the screening analysis and allows for a more granular understanding of the intensity of the main and interaction effects. The visualization demonstrates that factors such as dynamic viscosity and liquid mass flow rate are the most critical in determining penetration length, both individually and in combination with other parameters. Meanwhile, parameters like surface tension play a less significant role.

## Conclusion

This work investigated the internal two-phase mixing in a flow-blurring (FB) injector by combining unsteady VOF simulations with a two-level $2^{5}$ Full-Factorial Design (FFD), 32 runs, using a RANS-based solver. Penetration length, flow morphology, and turbulence statistics were evaluated over air-to-liquid mass flow rate ratios (ALRs). The main quantitative findings are summarised below:

- **Flow Morphology:** The analysis provided valuable insights into the flow regimes, particularly air-dominant, bubbly flow, and liquid-dominant regimes. These regimes significantly influence the flow properties inside the liquid tube and near the injector outlet. The study revealed distinct volume fraction distributions and flow structures depending on the regime, with higher air momentum penetrating the liquid tube in the air-dominant and bubbly regimes, while in the liquid-dominant regime, the air’s momentum was insufficient to cause significant penetration. In total, penetration length ranged from $2.0$ to $8.5$ [mm] over the simulated cases.
- **Screening Analysis:** The screening analysis focused on the main effects and two-way interactions of five input factors, namely liquid density, dynamic viscosity, surface tension, liquid mass flow rate, and air mass flow rate. The results identified dynamic viscosity and liquid mass flow rate as the most influential factors affecting penetration length, with dynamic viscosity showing the strongest inverse relationship. Surface tension, while contributing to the penetration dynamics, had a less significant impact compared to other factors.
- **Correlation Analysis:** This analysis provided a quantitative understanding of the main effects and two-way interactions. The analysis validated the screening results by confirming that liquid mass flow rate and dynamic viscosity exert strong negative correlations with penetration length, while air mass flow rate has a positive effect. The two-way interactions, such as those between liquid and air mass flow rates, demonstrated that higher air mass flow could counterbalance the negative effects of increased liquid flow or viscosity.

The results of this study provide a comprehensive understanding of the factors and interactions affecting flow-blurring atomization and penetration length. These insights are critical for optimizing the design and performance of flow-blurring injectors across various applications, including combustion and spray cooling systems.

Although the present study provides important parametric insights into flow-blurring injectors, several limitations should be acknowledged. First, the simulations are based on a RANS approach with wall functions, which may underpredict fine-scale turbulence effects and interfacial instabilities, particularly in the near-nozzle region. Second, the current configuration considers ambient pressure and does not account for compressibility or elevated-temperature effects relevant to combustion applications. Third, while a range of liquid properties is covered, the model does not accurately incorporate cavitation or phase change phenomena.

Future studies should also aim to incorporate machine learning models to predict penetration length and flow dynamics based on the insights from this study. Using regression models such as Multilayer Perceptron (MLP) and Convolutional Neural Networks (CNN), future work could generate a more efficient predictive framework that can optimize the input parameters for specific applications. In recent years, Physics-Informed Neural Network (PINN) algorithms have also shown great success in different CFD applications, including the work of by the last author^47,48^ providing another potential pathway to extend the present work and obtain optimise input parameters. Furthermore, experimental validation of the numerical findings will be essential to ensure the generalizability of the proposed models across different operational conditions.

## Data Availability

The datasets used and/or analyzed during the current study are available from the corresponding author upon reasonable request.
